# Left and right atrial strain analysis to predicting new-onset atrial fibrillation in patients with septic shock: a single-center retrospective echocardiography study

**DOI:** 10.1186/s13054-024-05024-9

**Published:** 2024-07-11

**Authors:** Christophe Beyls, Alexis Hermida, Camille Daumin, Max-Paul Delmotte, Arnaud Nsiku, Pierre Huette, Camille Bunelle, Hervé Dupont, Osama Abou-Arab, Yazine Mahjoub

**Affiliations:** 1 Department of Anesthesiology and Critical Care Medicine, Amiens University Hospital, 1, rond point du Pr Cabrol, 80054 Amiens Cedex 1, France; 2 UR UPJV 7518 SSPC (Simplification of Care of Complex Surgical Patients) Research Unit, University of Picardie Jules Verne, Amiens, France; 3 Department of Cardiology. Rythmology Unit, Amiens University Hospital, 80054 Amiens, France

To the Editor,

New-onset atrial fibrillation (NOAF) is the most common arrhythmogenic complication in septic shock patients, significantly increasing the risk of thromboembolic events and mortality [1]. Septic shock may rapidly induce septic cardiomyopathy, leading to atrial remodeling and fibrosis, which predisposes patients to NOAF. Although bedside transthoracic echocardiography (TTE) is commonly used to assess cardiac function, no echocardiographic parameters have been specifically linked to NOAF in septic shock patients. Speckle tracking strain analysis is a novel method for evaluating the physiological phases of the left atrium (LA) and the right atrium (RA). LA dysfunction, mainly assessed through the reservoir phase (LASr), has been associated with NOAF in various diseases [2]. Recent findings suggest that the RA reservoir phase (RASr) is also beneficial for identifying high-risk NOAF patients [3]. However, no study has combined LASr and RASr analyses to predict NOAF in septic patients. We aim to explore the diagnostic ability of LASr and RASr for identifying patients at high risk of NOAF, their association with NOAF occurrence, and their correlation with 30-day mortality.

We conducted a retrospective analysis of a prospective echocardiographic database (ATRIALSEPSIS registry, PI2021_843_0179) of adult patients admitted to our intensive care unit (ICU) at Amiens University Hospital for septic shock, as defined by Sepsis-3 criteria. All patients underwent a TTE in sinus rhythm within 48 h of admission. Exclusion criteria included a history of AF, AF during TTE, permanent pacing, cardiac assist devices, and poor image quality for LAS analysis.

The primary endpoint was the occurrence of NOAF during ICU stay. The secondary outcome was the association between NOAF and 30-day all-cause mortality. TTE was performed within 48 h of ICU admission. Strain measurements (left and right ventricular and LASr) were conducted using dedicated automated software (Auto-Strain QLAB 15.0, Philips Medical Systems). At the time of the study, no dedicated software was available for RASr analysis. LASr and RASr were measured according to international guidelines [2]. A sample size of 126 patients was calculated to ensure over 95% power to detect a 1 0-unit difference in LASr between groups at a 5% significance level.

Given the known incidence of NOAF in septic shock (30%) and an expected clinical difference of 10 units in LASr between groups, this sample size provides over 95% power to detect a significant difference at a 5% significance level.

Between August 2021 and April 2023, 156 patients met the inclusion criteria. Thirty were excluded: 22 (14%) for poor TTE image quality, 6 (4%) for a history of AF, and 2 (1%) for developing NOAF before TTE. Ultimately, 126 patients were included, 40 (32%) developing NOAF and 86 (68%) without NOAF. There were no significant differences in age, SAPS II, medical history, or septic shock etiology between groups. NOAF appeared two days (median) post-TTE, requiring electrical cardioversion in 30% of cases and lasting a median of 14 h. LASr and RASr were impaired in the NOAF group (20.0% vs. 33.5%; p < 0.001 and 28.9% vs. 43.0%; p < 0.001, respectively). There was no difference in 30-day mortality (28% vs. 27%; p = 1). The LASr ROC curve had the highest AUC (0.76) for identifying NOAF, with a threshold of 20%, compared to a threshold of 30% for RASr (AUC 0.75, Fig. 1A). There was no significant difference between the AUC values (p = 0.09). LAS measurement feasibility was 94%, and RAS was 81%. The ICC parameters were > 0.7, indicating good reproducibility.

**Fig. 1:**
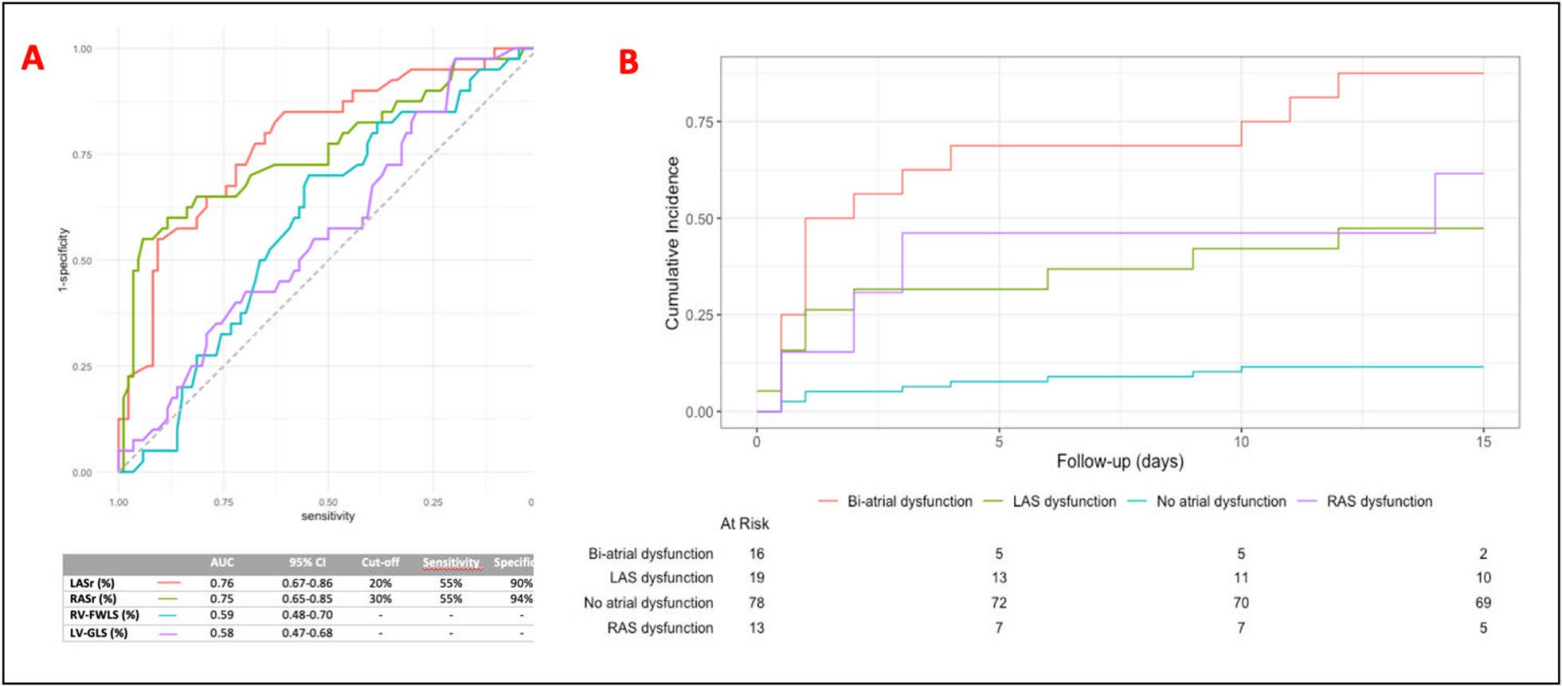
**A** ROC curve analysis of four-cardiac chambers strain parameters for prediction NOAF; and **B** Cumulative risk of NOAF according to the presence of LA, RA, or bi-atrial dysfunction. LAS: left atrial strain; GLS-4ch: global longitudinal strain on a four-chamber apical view; NOAF: new-onset atrial fibrillation; RAS: right atrial strain; RV-FWLS: right ventricular free wall longitudinal strain

In a multivariable analysis using nested models, LA dysfunction, defined by a LASr < 20%, was strongly associated with NOAF (OR = 10.48; p = 0.003). Adding RA dysfunction, defined as a RASr < 30%, improved model discrimination and was also strongly associated with NOAF (OR = 8.28; p = 0.004). Patients with bi-atrial dysfunction (LASr < 21% and RASr < 30%) had a 80% cumulative risk of developing NOAF within 15 days, compared to 11% in those without (p < 0.0001, Fig. 1B). NOAF, LA, and RA dysfunction were not associated with 30-day mortality.

Impairment of LASr function is a recently discovered aspect of atrial cardiomyopathy associated with NOAF in various pathologies [2, 4]. In septic shock patients, septic cardiomyopathy affects ventricular function [5], but studies on atrial myocardium are lacking. Although research has focused on LA, RA is also an arrhythmogenic substrate for NOAF, supporting the understanding that AF is a bi-atrial disease [3].

This is the first study to analyze LASr and RASr in a significant cohort of septic shock patients, showing that both reservoir phases were impaired and emphasizing the importance of assessing LA and RA functions to predict NOAF.

Our study has limitations due to its retrospective design. We mitigated biases by calculating the necessary sample size for statistical significance. Moreover, strain measurements required advanced echocardiographic machines and expertise. Nevertheless, AI development for automatic measurements will aid its broader use. Finally, consistent follow-up with the same software is recommended because LASr and RASr values depend on the software and version.

To conclude, we found that in the early phase of septic shock, patients with biatrial dysfunction assessed by atrial strain analysis are highly likely to develop NOAF. Early identification of these patients may enable personalized treatment selection to reduce the likelihood of NOAF.

## Data Availability

The datasets used and/or analyzed during the current study are available from the corresponding author on reasonable request.

## Abbreviations

ICU: Intensive care unit
LA: Left atrial
LAS: Left atrial strain
LASr: Left atrial strain during the reservoir phase
NOAF: New-onset atrial fibrillation
RA: Right atrial
RASr: Right atrial strain during the reservoir phase
TTE: Transthoracique echocardiography
